# Supplementary figures and images for: LPS-induced systemic inflammation is suppressed by the PDZ motif peptide of ZO-1 via regulation of macrophage M1/M2 polarization
Source: eLife. 2024 Oct 8;13:RP95285. doi: 10.7554/eLife.95285 (PMC11460976; doi:10.7554/eLife.95285)

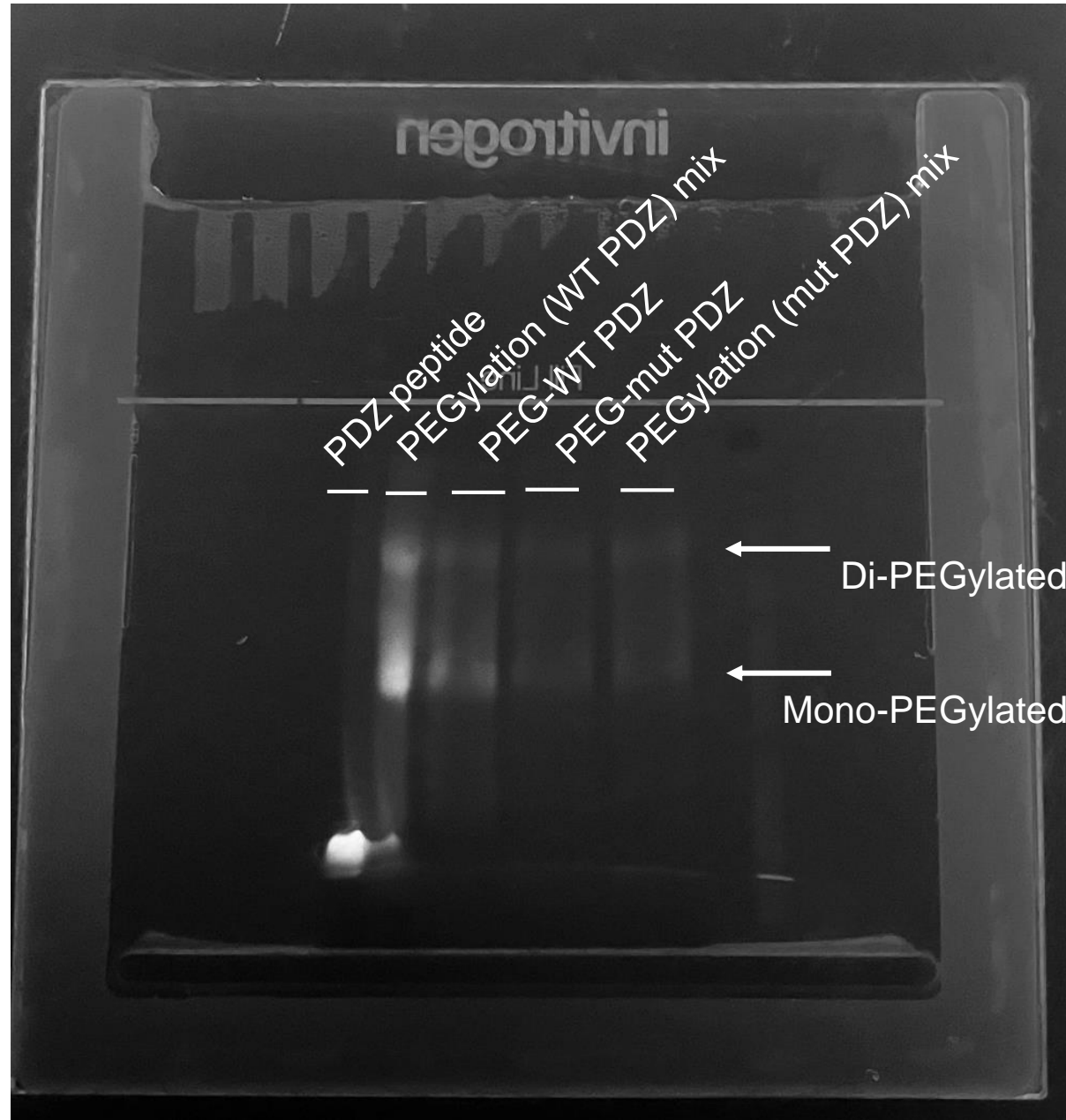

Supplement: Figure 1—source data 1. [file elife-95285-fig1-data1.pdf]

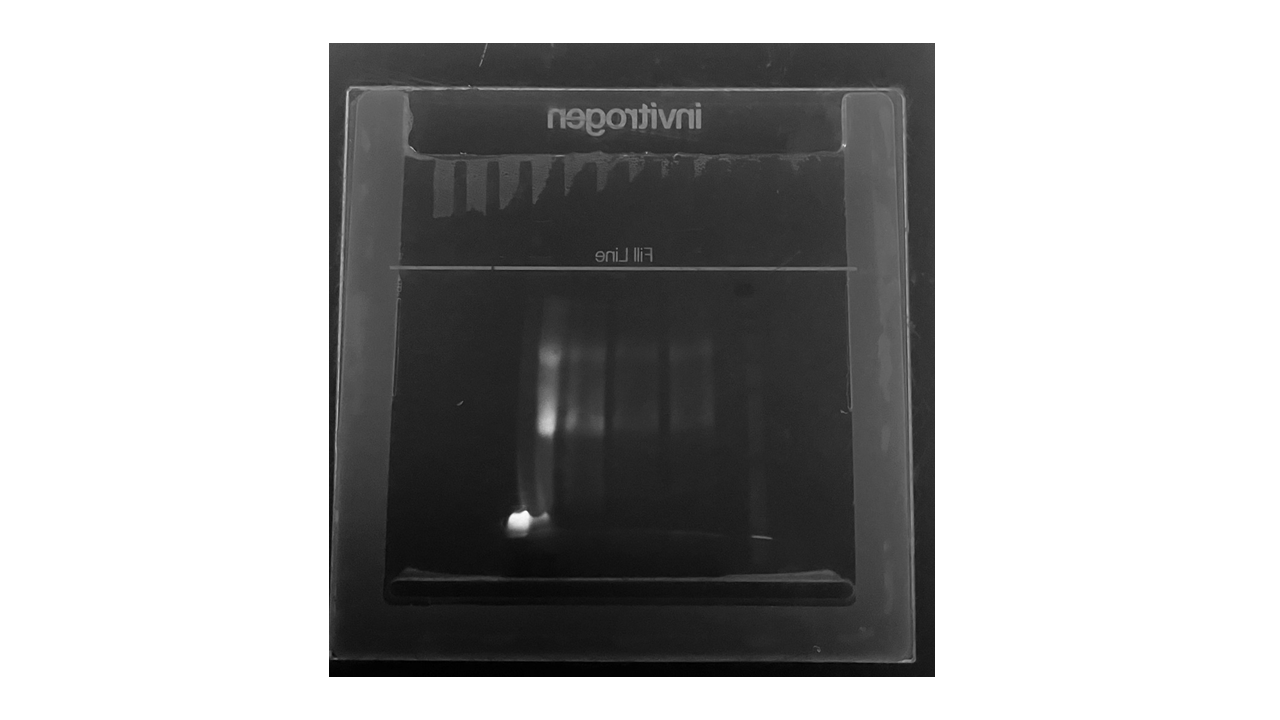

Supplement: Figure 1—source data 2. [file elife-95285-fig1-data2.tif]

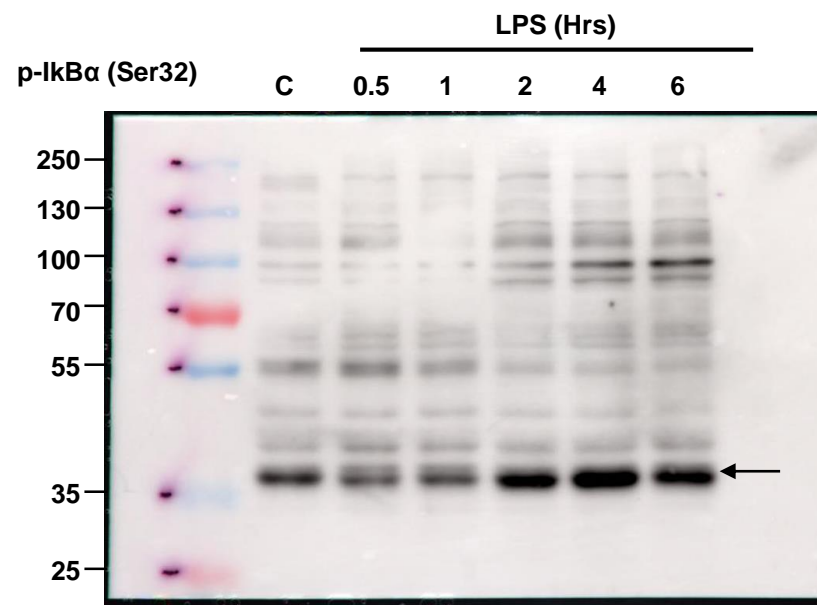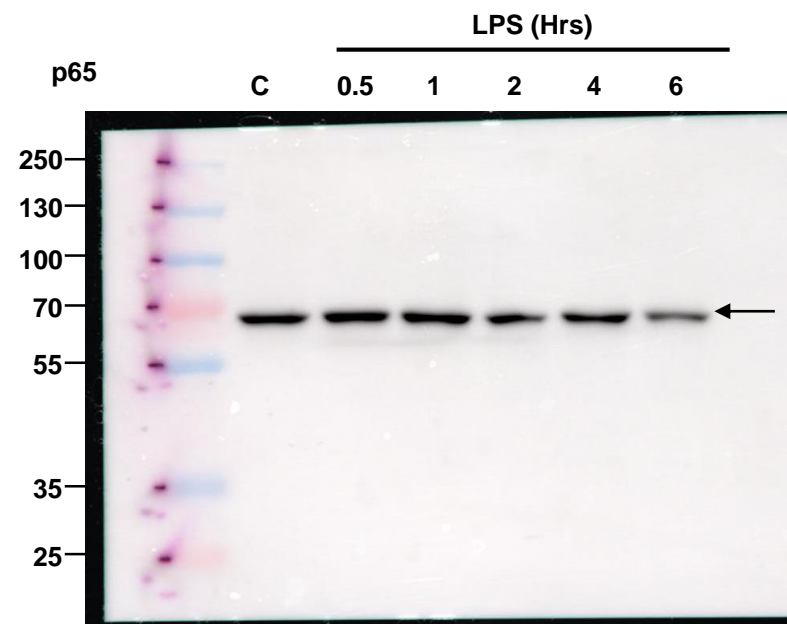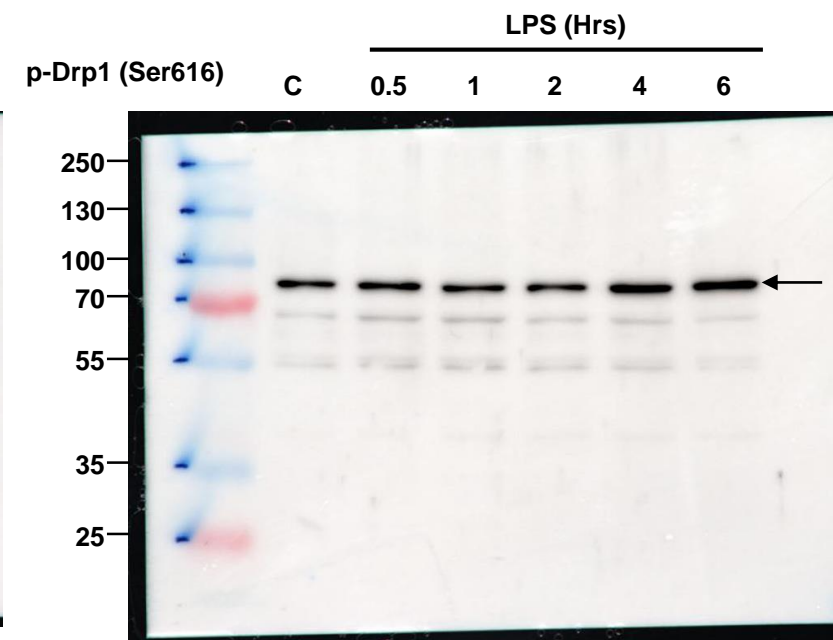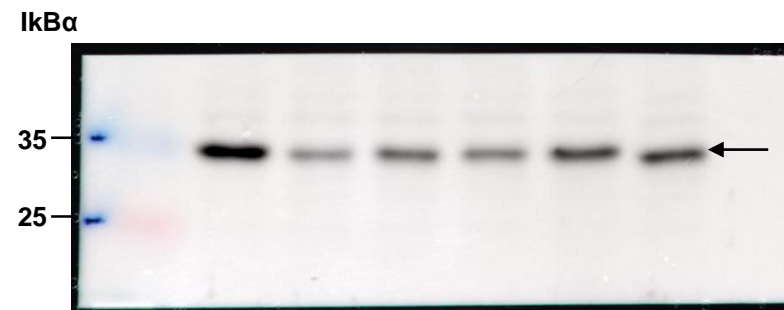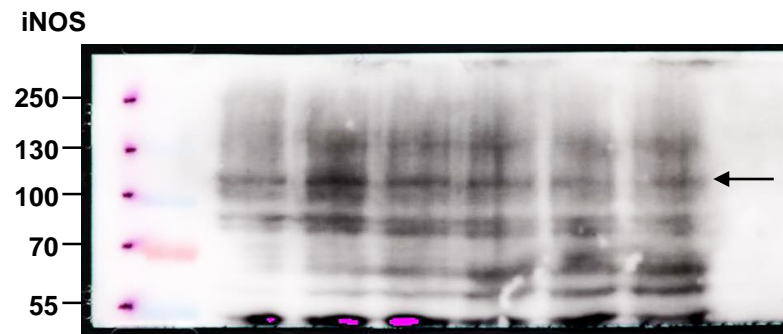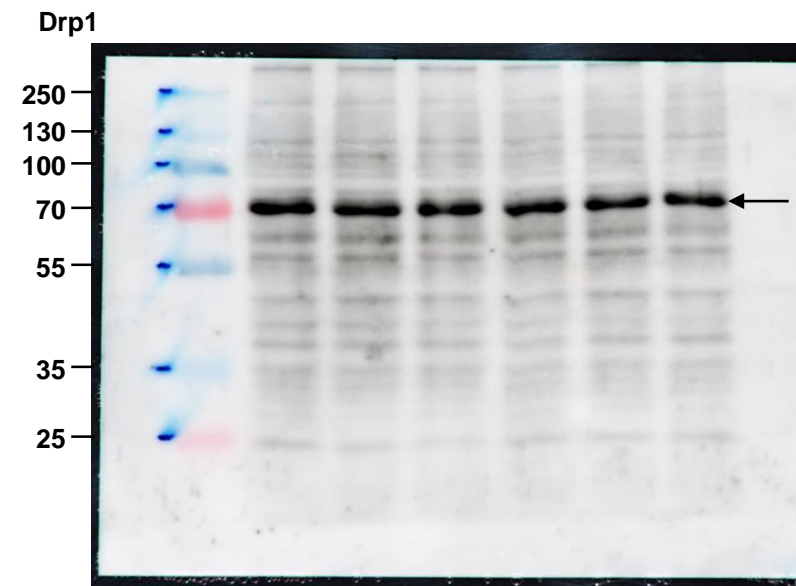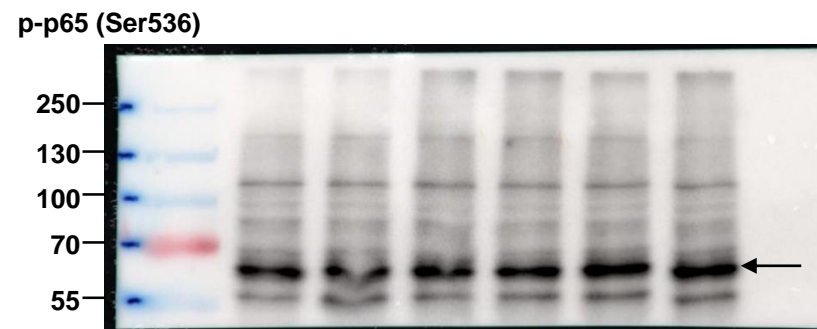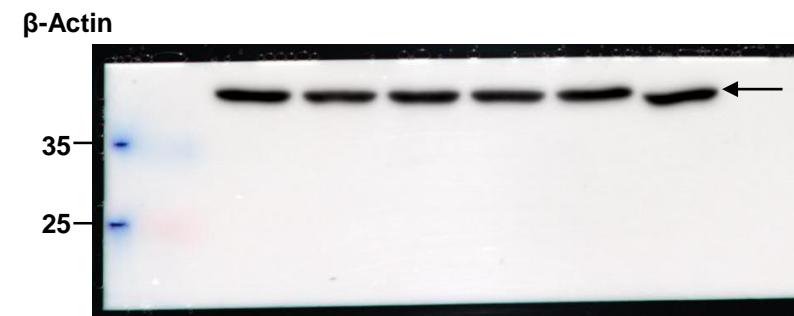

Supplement: Figure 6—source data 1. [file elife-95285-fig6-data1.zip › Figure6SourceData1/Figure 6-Source data 1 Uncropped and labeled gels for Figure 6.pdf]

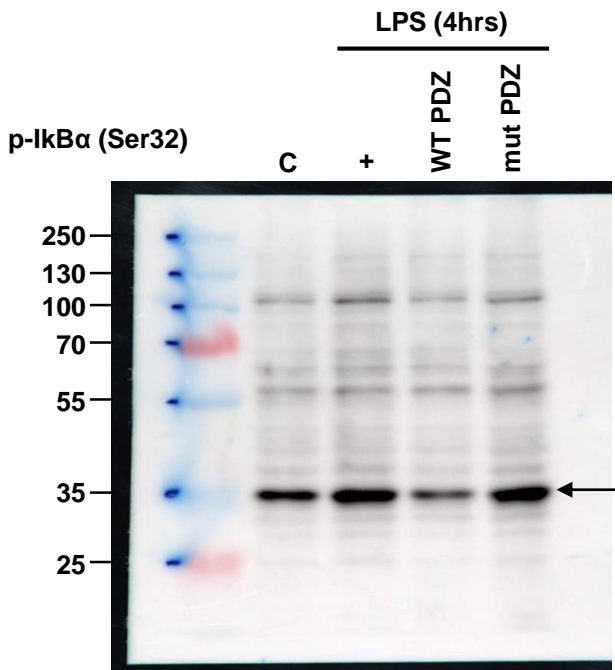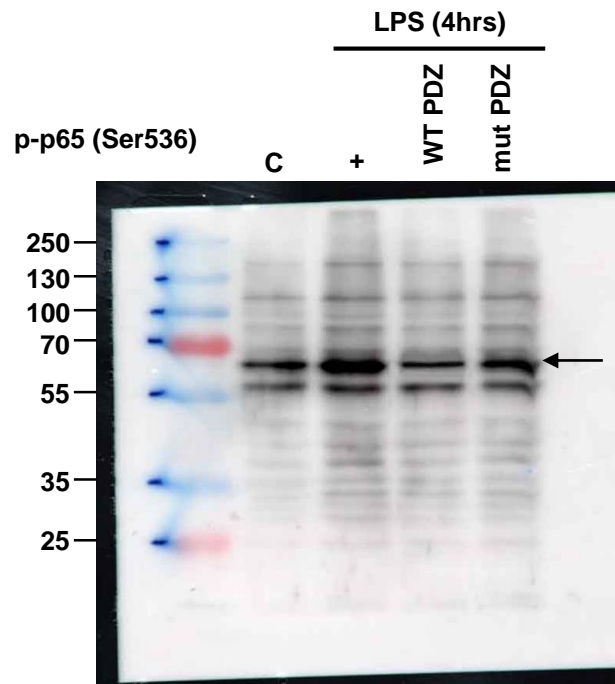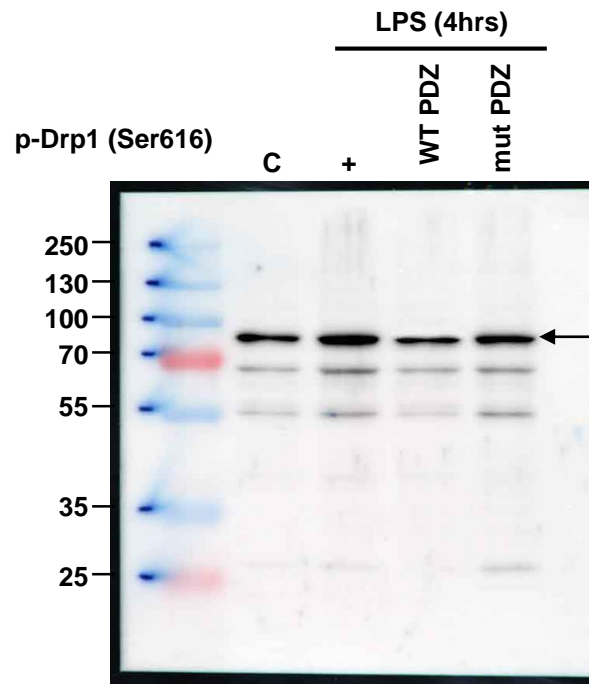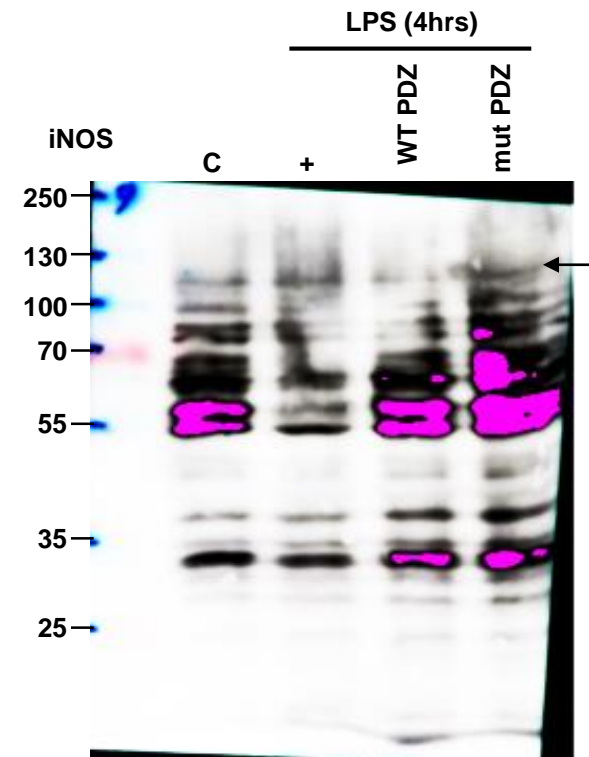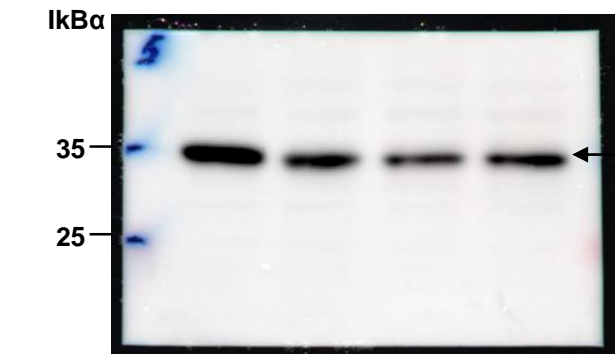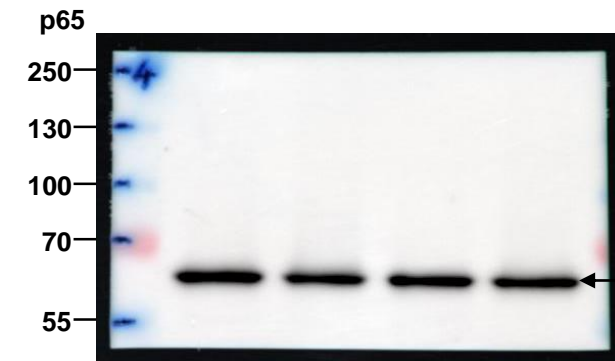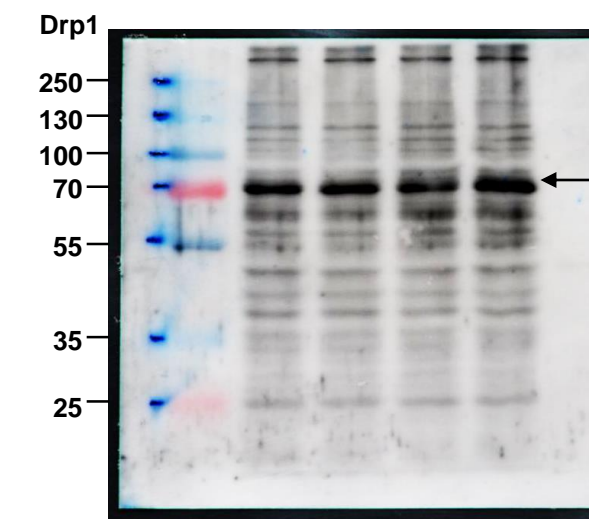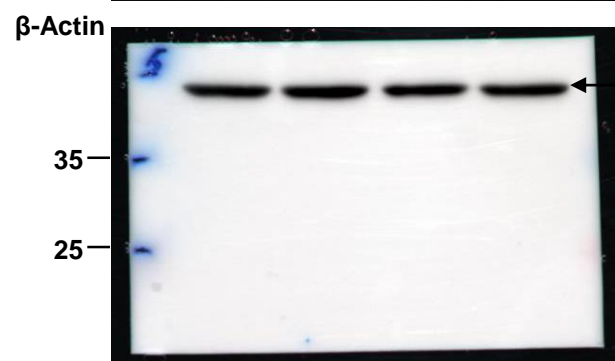

Supplement: Figure 6—source data 1. [file elife-95285-fig6-data1.zip › Figure6SourceData1/Figure 6-Source data 2 Uncropped and labeled gels for Figure 6.pdf]

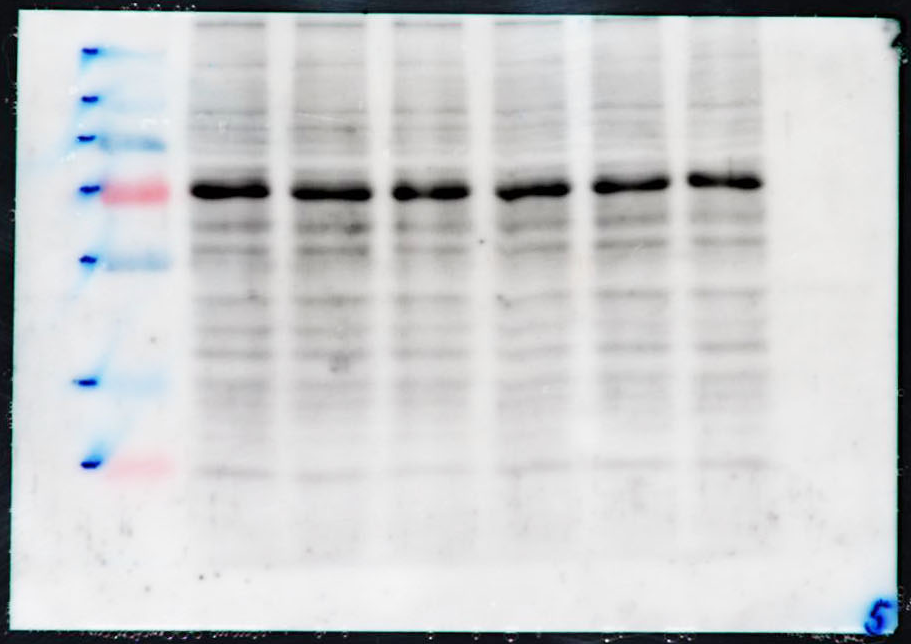

Supplement: Figure 6—source data 2. [file elife-95285-fig6-data2.zip › Figure6SourceData2/Figure 6-Source data 7 Raw unedited gels for Drp1.tif]

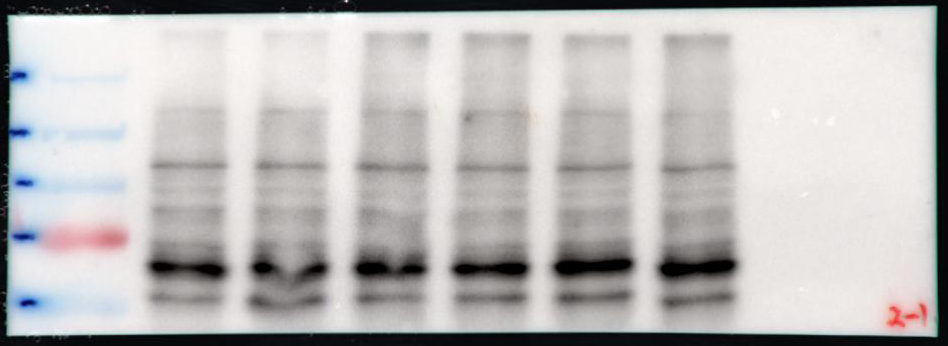

Supplement: Figure 6—source data 2. [file elife-95285-fig6-data2.zip › Figure6SourceData2/Figure 6-Source data 3 Raw unedited gels for p-p65 (Ser536).tif]

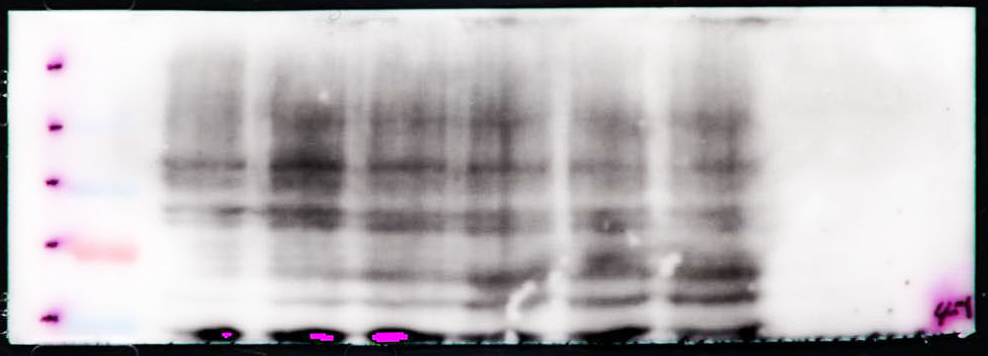

Supplement: Figure 6—source data 2. [file elife-95285-fig6-data2.zip › Figure6SourceData2/Figure 6-Source data 5 Raw unedited gels for iNOS.tif]

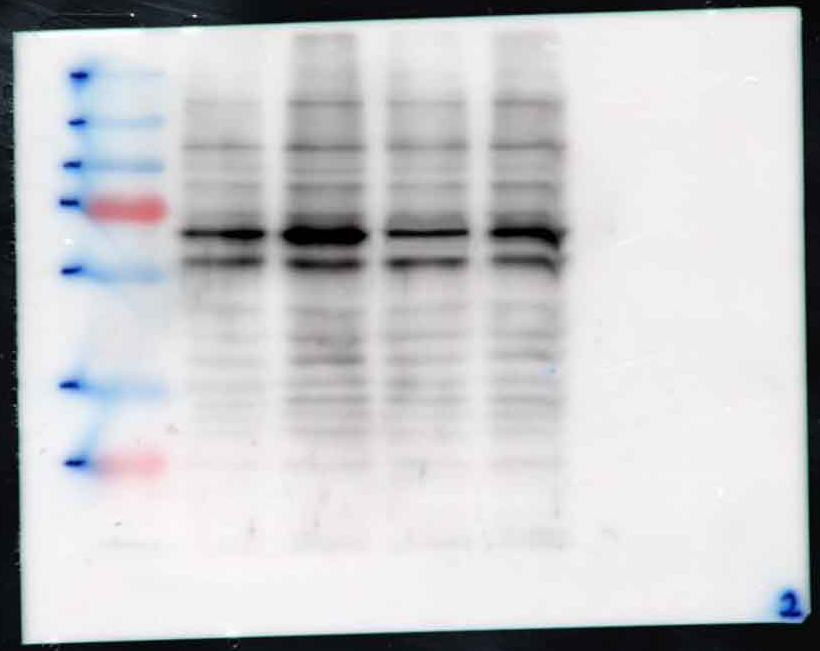

Supplement: Figure 6—source data 2. [file elife-95285-fig6-data2.zip › Figure6SourceData2/Figure 6-Source data 11 Raw unedited gels for p-p65 (Ser536).tif]

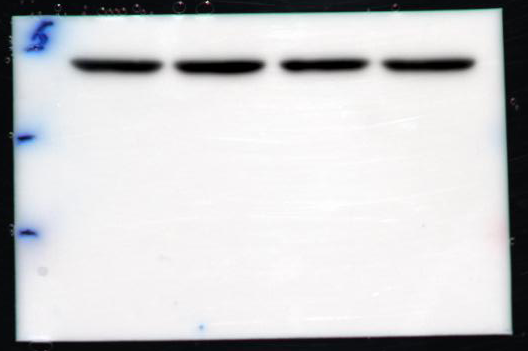

Supplement: Figure 6—source data 2. [file elife-95285-fig6-data2.zip › Figure6SourceData2/Figure 6-Source data 16 Raw unedited gels for actin.tif]

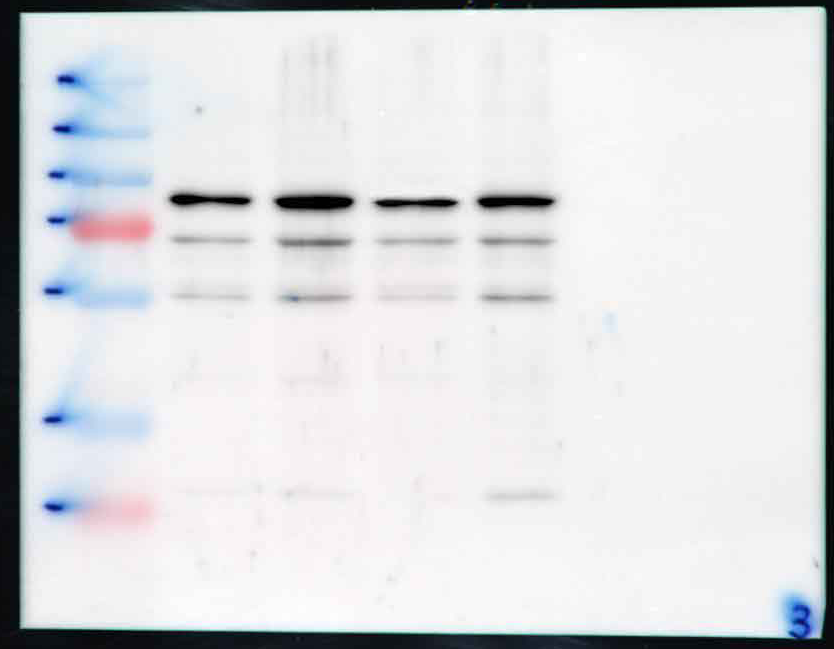

Supplement: Figure 6—source data 2. [file elife-95285-fig6-data2.zip › Figure6SourceData2/Figure 6-Source data 14 Raw unedited gels for p-Drp1 (Ser616).tif]

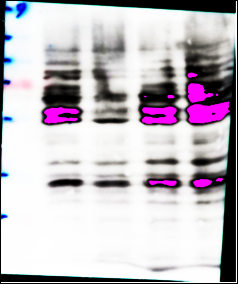

Supplement: Figure 6—source data 2. [file elife-95285-fig6-data2.zip › Figure6SourceData2/Figure 6-Source data 13 Raw unedited gels for iNOS.tif]

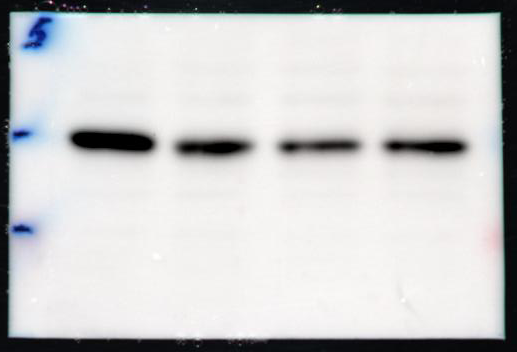

Supplement: Figure 6—source data 2. [file elife-95285-fig6-data2.zip › Figure6SourceData2/Figure 6-Source data 10 Raw unedited gels for IkB.tif]

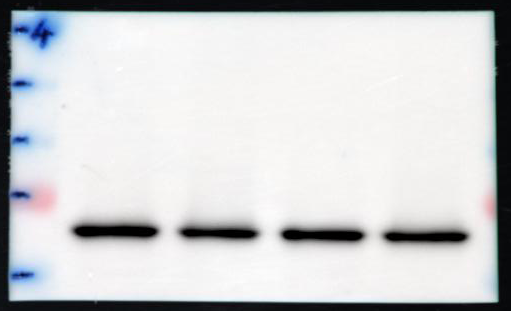

Supplement: Figure 6—source data 2. [file elife-95285-fig6-data2.zip › Figure6SourceData2/Figure 6-Source data 12 Raw unedited gels for p65.tif]

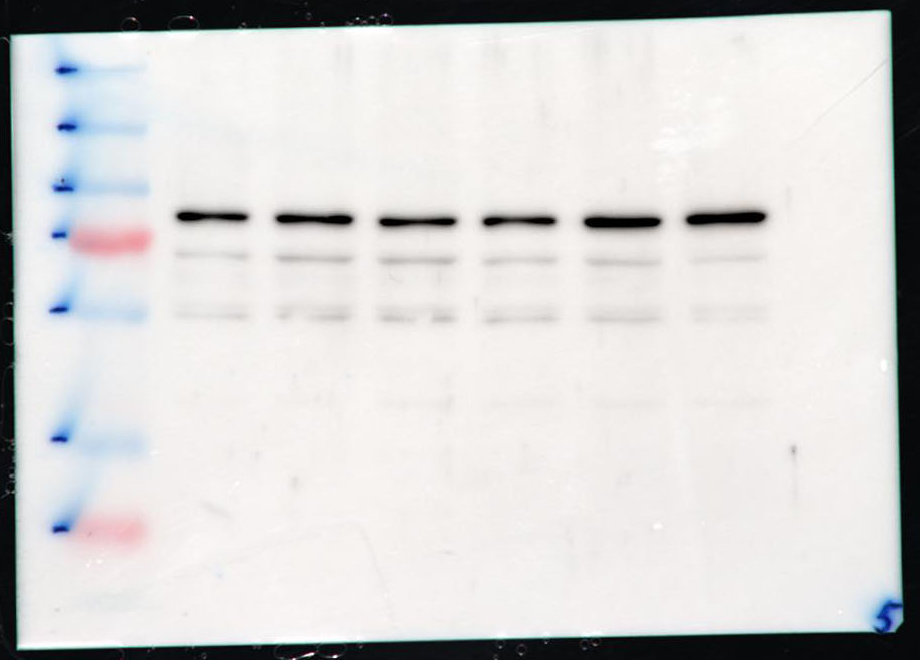

Supplement: Figure 6—source data 2. [file elife-95285-fig6-data2.zip › Figure6SourceData2/Figure 6-Source data 6 Raw unedited gels for p-Drp1 (Ser616).tif]

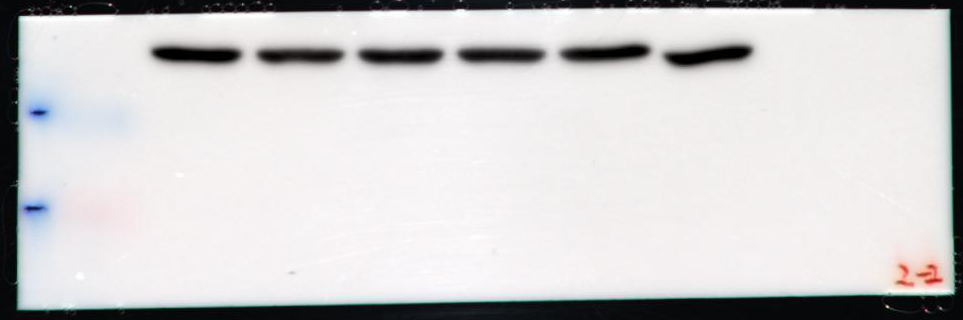

Supplement: Figure 6—source data 2. [file elife-95285-fig6-data2.zip › Figure6SourceData2/Figure 6-Source data 8 Raw unedited gels for actin.tif]

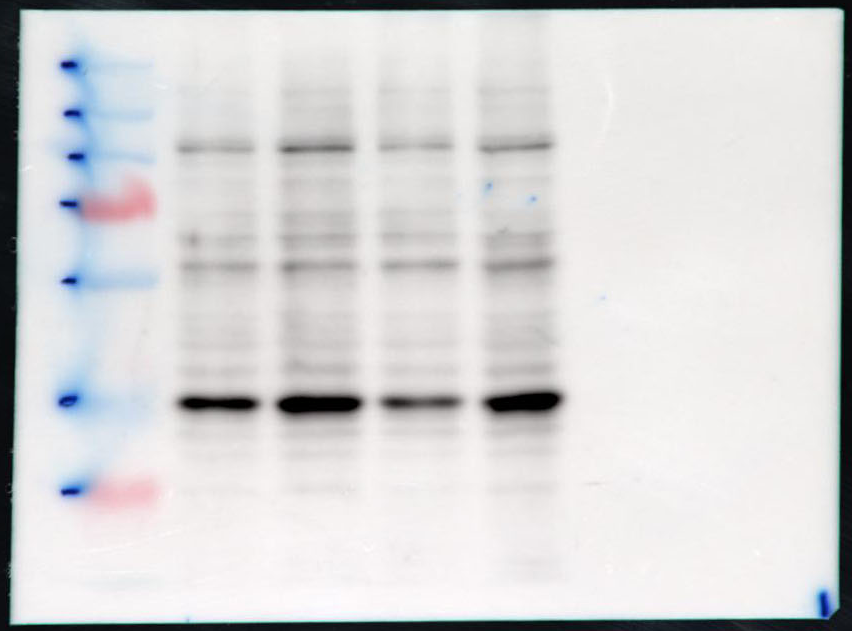

Supplement: Figure 6—source data 2. [file elife-95285-fig6-data2.zip › Figure6SourceData2/Figure 6-Source data 9 Raw unedited gels for p-IkB (Ser32).tif]

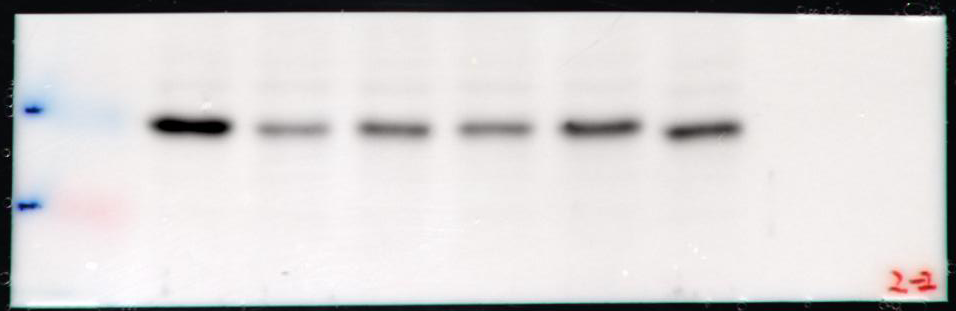

Supplement: Figure 6—source data 2. [file elife-95285-fig6-data2.zip › Figure6SourceData2/Figure 6-Source data 2 Raw unedited gels for IkB.tif]

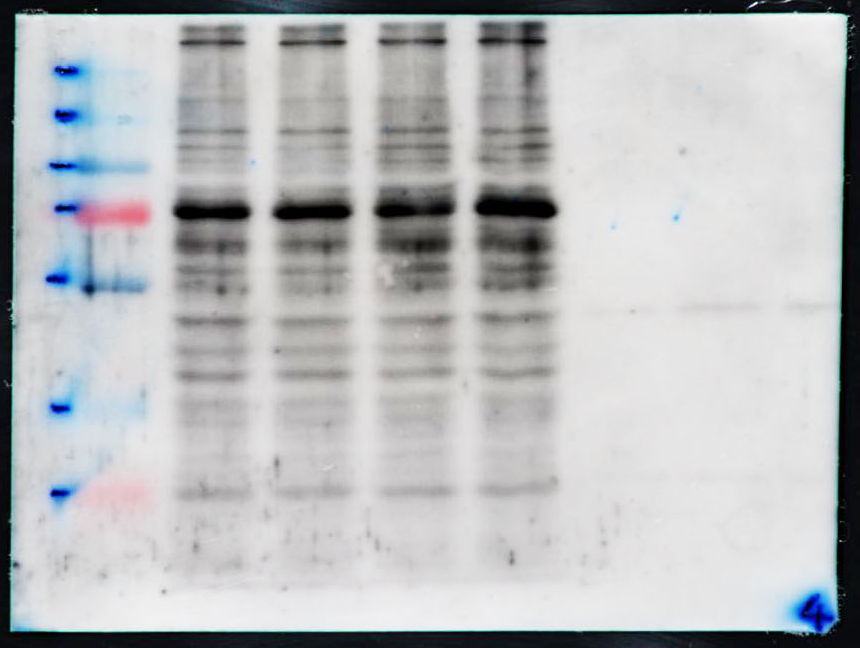

Supplement: Figure 6—source data 2. [file elife-95285-fig6-data2.zip › Figure6SourceData2/Figure 6-Source data 15 Raw unedited gels for Drp1.tif]

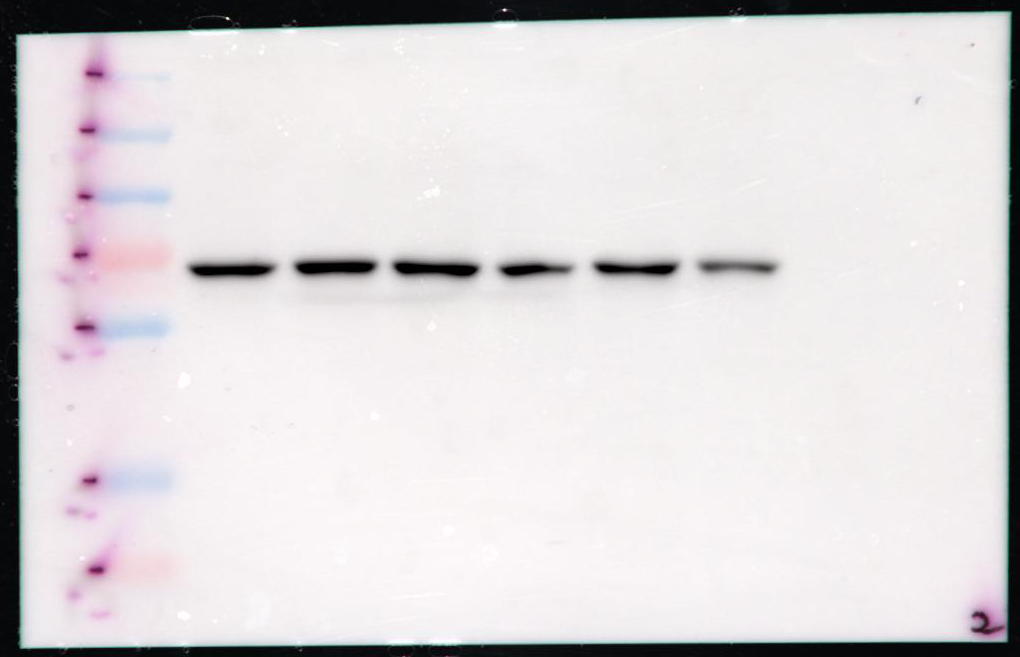

Supplement: Figure 6—source data 2. [file elife-95285-fig6-data2.zip › Figure6SourceData2/Figure 6-Source data 4 Raw unedited gels for p65.tif]

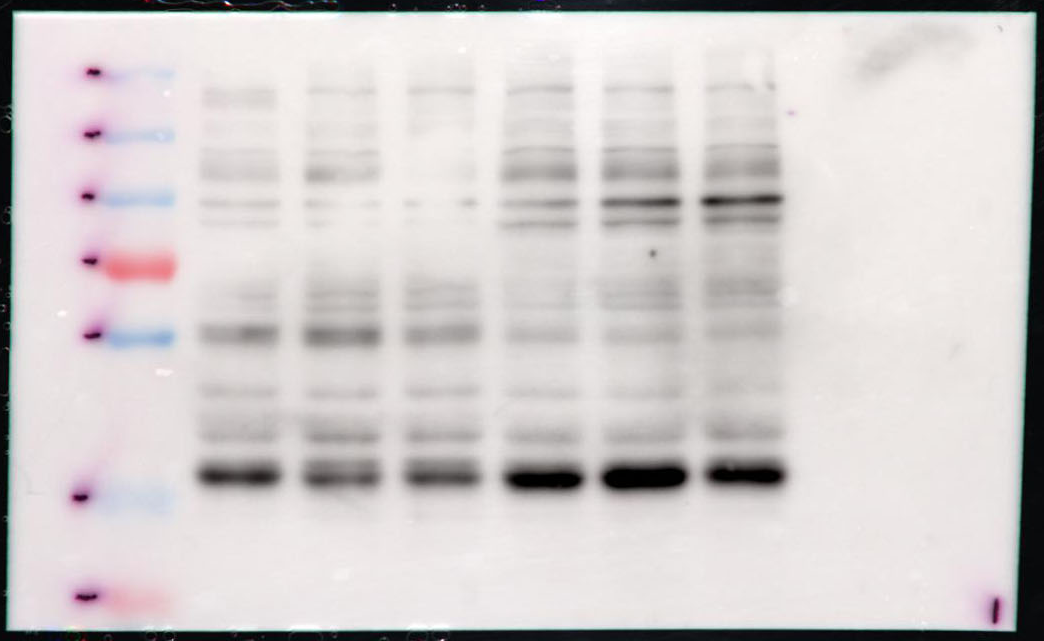

Supplement: Figure 6—source data 2. [file elife-95285-fig6-data2.zip › Figure6SourceData2/Figure 6-Source data 1 Raw unedited gels for p-IkB (Ser32).tif]
